# What if consciousness is not an emergent property of the brain? Observational and empirical challenges to materialistic models

**DOI:** 10.3389/fpsyg.2022.955594

**Published:** 2022-09-07

**Authors:** Helané Wahbeh, Dean Radin, Cedric Cannard, Arnaud Delorme

**Affiliations:** ^1^Research Department, Institute of Noetic Sciences, Petaluma, CA, United States; ^2^Swartz Center for Computational Neuroscience, Institute of Neural Computation, University of California, San Diego, San Diego, CA, United States

**Keywords:** non-local consciousness, orch-OR, integrated information theory, global work-space theories, higher-order theories, re-entry and predictive processing, analytic idealism, interface theory of perception

## Abstract

The nature of consciousness is considered one of science’s most perplexing and persistent mysteries. We all know the subjective experience of consciousness, but where does it arise? What is its purpose? What are its full capacities? The assumption within today’s neuroscience is that all aspects of consciousness arise solely from interactions among neurons in the brain. However, the origin and mechanisms of *qualia* (i.e., subjective or phenomenological experience) are not understood. David Chalmers coined the term “the hard problem” to describe the difficulties in elucidating the origins of subjectivity from the point of view of reductive materialism. We propose that the hard problem arises because one or more assumptions within a materialistic worldview are either wrong or incomplete. If consciousness entails more than the activity of neurons, then we can contemplate new ways of thinking about the hard problem. This review examines phenomena that apparently contradict the notion that consciousness is exclusively dependent on brain activity, including phenomena where consciousness appears to extend beyond the physical brain and body in both space and time. The mechanisms underlying these “non-local” properties are vaguely suggestive of quantum entanglement in physics, but how such effects might manifest remains highly speculative. The existence of these non-local effects appears to support the proposal that post-materialistic models of consciousness may be required to break the conceptual impasse presented by the hard problem of consciousness.

## What is consciousness?

The term “consciousness” means different things to different audiences. From a lay perspective, the fact of consciousness (here meaning *awareness*) is so self-evident that the only question that may arise is why anyone would consider consciousness to be mysterious in the first place, akin to asking a fish, “What is water?” From a scientific perspective, the unsolved mystery is how consciousness emerges from brain activity. How does a three-pound lump of tissue inside the skull give rise to a mind that is self-aware and enjoys subjective experience? In philosophy, which has debated the “mind-body problem” for millennia, many sets of assumptions have been proposed, ranging from materialism (i.e., all is dependent on or reducible to the physical) to idealism (i.e., ideas or thoughts make up fundamental reality). For mystics and others entranced by the esoteric traditions, the problem is not so much about the mind but how the physical world emerges from a non-physical “substance.”

Over the past few decades, most scientifically oriented research on consciousness has studied consciousness as a controlled variable. They have reduced consciousness to simpler constructs, such as perception, and focused on comparisons in brain processes during conscious and unconscious conditions, the so-called “contrastive approach.” In this approach, differences in brain activity are examined when the same stimulus is subjectively perceived versus when it is not (Baars, 2005). This search for the neural correlates of consciousness (NCC) is defined as the “minimum neuronal mechanisms jointly sufficient for any one specific conscious experience” (Koch et al., 2016). Experimental designs have used methods like stimulus masking (Shapiro et al., 1997; Simons and Chabris, 1999; Dehaene et al., 2001) or binocular rivalry (Leopold and Logothetis, 1999) to examine the brain activity associated with subliminal perception where information is not consciously perceived yet processed by the brain. Other approaches have relied on brain lesions (Hebb and Penfield, 1940), or, more recently, by artificially modulating brain activity in specific regions and networks intracranially, during neurosurgery, or non-invasively with transcranial magnetic/electric/ultrasound stimulation (Selimbeyoglu and Parvizi, 2010). Other authors have focused solely on states of unconsciousness during general anesthesia (Hudetz, 2012), epilepsy (Blumenfeld and Taylor, 2003), or sleep (Steriade et al., 2001). While a front line of research, the NCC approach has conceptual limitations. Mainly, the terms “consciousness” and “correlates” are enmeshed because brain events that co-vary with conscious experience can either be the experience’s neural substrates, the prerequisites, or even the experience’s neural consequences (Aru et al., 2012; de Graaf et al., 2012).

Several scientific theories of consciousness are primed to be tested experimentally instead of merely identifying correlations between conscious/unconscious events and brain activity. In the sections below, we briefly summarize the main neuroscience theories, referred to as physicalist or reductionist theories, most of which assume that consciousness emerges from the brain. While most physicalist theories aim to explain different aspects of consciousness, they often share similarities and have been recently grouped into four categories: higher-order theories (HOTs), global work-space theories (GWTs), integrated information theory (IIT), and re-entry and predictive processing theories (Seth and Bayne, 2022). Here, we follow this categorization and present a brief summary. However, a full review of each theory is beyond this article’s scope (see Seth and Bayne, 2022, for a full review).

## Physicalist theories of consciousness

### Global work-space theories

Cognitive scientist Bernard J. Baars first proposed the GWT in 1983. GWT is a cognitive architecture inspired by artificial intelligence where a centralized resource is available through which specialized processors share and receive information (Baars, 2005; Baars et al., 2021). The theory is based on the observation that there are highly specialized brain regions that process information locally and unconsciously, such as the visual cortex. Conscious experience occurs once there is a distributed activity in other brain areas, that is, “broadcasting” to the system as a whole (Baars, 2005). The widespread access, operation, and coordination of specialized neural networks, which would otherwise operate autonomously, is coordinated by consciousness, involving mainly the frontoparietal network and high-frequency oscillatory rhythms (Baars, 2005).

Dehaene and colleagues have adjusted the GWT to account for new knowledge about the brain, the so-called “neuronal global work-space” (Dehaene et al., 2003). For example, global activity among work-space neurons is generated by excitatory neurons responding to sensory stimuli with long-range cortico-cortical connections. In turn, this global activity inhibits alternative activity patterns among the work-space neurons to prevent the conscious experience of other stimuli (e.g., attentional blink). This inhibition mechanism would allow the unified experience of consciousness (the composition and exclusion axioms in IIT). Supported by experimental findings in the context of the search for NCCs, this popular model suggests that (1) most of the brain’s computations are performed in a non-conscious operation mode and that (2) conscious access must be distinguished from selective attention, (3) conscious perception may be characterized by a non-linear function that ‘ignites’ a network of distributed areas (a gradual increase in stimulus visibility accompanied by a sudden transition of the neuronal work-space into a pattern, i.e., the broadcasting), and (4) the selected information gains access to additional computations for conscious perception such as temporary maintenance, global sharing, flexible routing (Dehaene et al., 2014). The model predicts that measures of complexity, long-distance correlation, and integration of brain signals should provide reliable indices of conscious processing and has clinical applications (e.g., sleep, coma, anesthesia; Dehaene et al., 2014; Mashour et al., 2020). Some investigators have recently attempted to generalize the GWT to brain-inspired artificial architectures by implementing the GWT into deep learning algorithms (VanRullen and Kanai, 2021). GWT is promising as a model. However, it is unclear what determines when information is broadcasted to the whole (e.g., threshold) and what discriminates different types of subjective experiences within the GWT theory.

### Higher-order theories

The HOTs family includes, for example, the self-organizing meta-representational account theory (SOMA; Cleeremans et al., 2020), the adversarial framework for probabilistic computation (Gershman, 2019), and the perceptual reality monitoring theory (Lau, 2019). In this view, consciousness is defined as a higher-order representation of lower-order representations. In other words, subjective experiences reflect higher brain orders like meta-representations, which have learned to describe and interpret the lower-order functions such as local modules specialized in processing specific information. In this sense, consciousness is the brain’s unconscious, embodied, enactive, non-conceptual theory about itself (Cleeremans et al., 2020). While Gehrman’s model interprets this view in terms of computations and algorithms, Lau’s view is in terms of belief and epistemic justification on a subjective level. The NCCs for the HOTs generally consist in anterior regions of the brain, like the prefrontal cortex, reflecting their involvement in complex cognitive functions (Lau and Rosenthal, 2011).

The primary limitation of both GWTs and HOTs is that they do not account for the phenomenal differences between distinct subjective experiences (Seth and Bayne, 2022). Furthermore, neither GWTs nor HOTs have addressed the adaptative and evolutionary role of conscious experience (i.e., embodiment and environmental embeddedness; Seth and Bayne, 2022).

### Integrated information theory

Integrated information theory is a mathematical approach based on phenomenology by first identifying the essential properties of consciousness, the so-called axioms: *intrinsic information* – each experience is specific, there is intrinsic information in the system that is associated with that experience that differs from alternative experiences, *information* – consciousness is composed of a specific set of specific phenomenal distinctions and is different from other possible experiences, *integration* – consciousness is unified with each experience being irreducible to non-interdependent components, and *exclusion –* consciousness is unique in content and spatio-temporal context (Tononi, 2015; Tononi et al., 2016). IIT infers the postulates or requirements for a physical system to be a physical substrate of consciousness from the axioms.

Integrated information refers to a system’s constituents that are discriminated by their respective information. The whole cannot be reduced to the information of each part, called the “causal-effect power” (Oizumi et al., 2014). These irreducible maxima of additional integrated information generated by the system as a whole compared to its parts are termed and quantified as Φ (“phi”) and affect the probability of its past and future states. The larger the Φ value, the more intrinsic cause-effect power the system has and the more conscious it is (Koch, 2018). Thus, any complex and interconnected physical system with these properties will have some level or quantity of consciousness, corresponding to the amount of intrinsic cause-effect power the substrate has. The content of a conscious experience is predicted to be structurally identical to the cause-effect structure of its physical substrate (Albantakis, 2020). So the more structurally complex the system is, the more structurally complex the experience is. IIT, therefore, provides a potential method to (1) assess whether a physical system constitutes a physical substrate of consciousness through its compliance with the postulates, (2) quantify the level of consciousness of that system, and (3) estimate its phenomenological structure in causal terms (Albantakis, 2020).

Unlike HOTs and GWT, progress has been made to assess the relevance of environmental embodiment for consciousness (i.e., consciously capturing the causal structure of a rich environment). For example, Haun and Tononi (2019) showed how this is useful for successfully navigating spacetime (Haun and Tononi, 2019). Albantakis et al. (2014) showed that this is an important driving force for organisms to develop highly integrated networks (“brains”), leading to an increase in their internal complexity (Albantakis et al., 2014). These concepts bring evolutionary context to the development of consciousness and the complexity of the brain.

### Re-entry and predictive processing approaches

Re-entry theories were developed from the idea that “we are fooled into thinking that we know what we are conscious of” (Lamme, 2010, p. 204). Therefore, introspective or behavioral observations make understanding the mind-brain relationship impossible. Thus, this approach removes intuitive or psychological notions of conscious experience from the study of consciousness (Lamme, 2006). In the local recurrency theory, conscious perception corresponds to top-down signaling (Lamme and Roelfsema, 2000; Lamme, 2006; Seth and Bayne, 2022). Consciousness emerges from simple localized recurrent, top-down processing within perceptual cortices, and frontal and parietal regions would be crucial to perceptual experience content, reasoning, and decision making (Lamme and Roelfsema, 2000; Lamme, 2006, 2010). Local recurrency theory is similar to GWTs, except that we do not know what we are conscious of and that it is about perception. In contrast, GWT is about access (Lamme, 2010).

Predictive processing approaches in computational neuroscience, such as the hierarchical generative model, consider the brain as a machine that matches bottom-up inputs with top-down expectations through cortical processing, aiming to minimize the error in these predictions (Clark, 2013). In this bi-directional model, top-down connections from higher levels encode the predictions in the lower levels. This fully explains the bottom-up signal and leaves only residual prediction errors propagating information forward in the system to update the following predictions (Huang and Rao, 2011; Clark, 2013). This predictive control function is termed “active inference” (Seth and Bayne, 2022).

The adaptive resonance theory (ART) was developed by Stephen Grossberg and Gail Carpenter to address the “stability-plasticity dilemma,” or how the brain learns so quickly and stably without forgetting past knowledge (Grossberg, 2013a). For ART, various brain processes are required, namely Consciousness, Learning, Expectation, Attention, Resonance, and Synchrony (the CLEARS processes). Top-down expectations (E) direct the focus of attention (A) across competitive features. When a match occurs between the expectation and what is perceived, a resonant synchronization (RS) occurs and generates attentional focus driving fast learning (L) of bottom-up, called “many-to-one maps,” and top-down, called “one-to-many maps” representations. This whole process is called “adaptive resonance.” Here, the focus of attention corresponds to the minimization of error in the prediction function. There is growing experimental data supporting these predictions, and some ART models are thought to explain and predict behavioral, anatomical, neurophysiological, and biochemical data (Grossberg, 2013b).

In brief, for this family of theories, perceptual experience is the brain’s best guess of its cause (minimization of the prediction error) through the exchange of top-down predictions and bottom-up prediction errors (Rao and Ballard, 1999; Friston, 2010; Hohwy, 2013; Seth and Bayne, 2022). For example, subjective emotions are considered to emerge from cognitive evaluations of physiological changes in the body and their causes (“constructed emotion” and “interoceptive inference”; Seth, 2013; Barrett, 2016).

### Summary of physicalist models

One common element across physicalist theories is the uncertainty reduction that results from allocating mechanisms to consciousness. The system must settle into one unified and highly informative representational state (Hohwy and Seth, 2020). This point of uncertainty reduction often corresponds to a threshold at which the contents become conscious (e.g., broadcasting for GWT, optimization of signal-to-noise ratio for HOTs, φ for IIT, information integration, and learning for re-entry and predictive theories). The second common element is the high importance of top-down signaling (e.g., a system with no top-down dimension has no φ in IIT; Oizumi et al., 2014).

The first disagreement between these theories regards the distinction between consciousness and cognition. Cognitive access relies on consciousness in GWT, consciousness is cognitively accessible in HOTs, whereas cognition is possible without consciousness for IIT and vice versa for predictive processing and re-entry theories. The second, more critical, disagreement between physicalist theories is in regards to the unity of consciousness, i.e., the subjective experience of awareness that fully captures what it is like to be an agent at any time. It is required by IIT and may be supported by the broadcast in GWTs but is ignored by HOTs, re-entry, and predictive theories that do not consider this concept necessary. These various theories may, in fact, address different aspects of consciousness.

In the well-known parable, “The Blind Men and the Elephant,” each person attempts to describe the elephant but only touches one small part (Saxe, 2016). Thus, they arrive at very different conclusions about what an elephant is like: a tree, a fan, a rope, a spear. They commence arguing, each one convinced that they are correct in their conclusions. The story’s moral is that we must step back to observe broader perspectives to describe and fully understand the elephant’s nature. Similarly, these physicalist theories may describe some aspects of consciousness very well, but they likely do not describe it completely.

In conclusion, while extensive and rigorous efforts have attempted to find, test, and validate physicalist theories or NCCs (Reardon, 2019; Templeton World Charity Foundation, 2022), the field is far from consensus about which theories are valid and could potentially explain consciousness or neural differences between different phenomenological experiences. We suggest that the gaps in physicalist theories in explaining consciousness may arise because the debate is framed around how the brain *generates* consciousness. The theories discussed so far attempt to explain phenomenological experience or qualia through reductionist brain mechanisms or correlations, often equated to computational or information processing systems. Some of these theories (e.g., re-entry or predictive processing theories) even consider subjective reports and introspection to be unreliable. Consequently, none of these theories have completely and convincingly explained the nature of consciousness.

## A different approach: Non-local consciousness theories

Alternative non-physicalist theories may inform other aspects of consciousness that are not completely explained by physicalist theories. Physicalist theories usually assume that consciousness is generated solely and purely from the brain and is only local to the brain. Alternatively, non-physicalist theories do not make these assumptions, even though both types of theories attempt to explain the underlying brain mechanisms of consciousness. Physicalist theories purport that consciousness originates from physical substrates like neurons that have evolved to be more and more complex over time through adaptation, leading to the emergence of consciousness. Non-physical models do not assume a physical substrate generates consciousness, and many even propose that consciousness is, in fact, more fundamental than matter and spacetime. In this view, that is the natural view for most ancient and eastern cultures, matter and spacetime arise from consciousness rather than the other way around. Perhaps a non-physicalist framework where consciousness is considered fundamental and has non-local properties (such as at the quantum scale) would better explain the full range of reported human phenomenology. For example, there are well-documented experiences of people perceiving information from distant locations, the future, and mental impressions from other people without the use of rationale or traditional means (Cardeña, 2018). In addition, there are verified cases of cognitive function when the neural substrate is severely degenerated, precluding normal brain function. These experiences, most of which are currently regarded as anomalous, will be described in the Section “Phenomena suggested by a model of nonlocal consciousness” as cases of what would be observed should non-physicalist theories of consciousness be valid.

These and other documented phenomenological experiences suggest a different nature of consciousness: one that may not be exclusively generated by neuronal activity and exhibits properties that transcend the conventional constraints of spacetime and, therefore, the physical body. The term “non-local consciousness” has been proposed to denote these purported transcendent properties of consciousness (Dossey, 1994). Physicalist scientists typically consider such experiences anomalous because they challenge prevailing assumptions about the nature and role of consciousness in physical reality. The term non-local is also referenced as a central idea within physics as an aspect of the physical world. For example, the brain operating, even to a small extent, in a quantum fashion might be a valid explanation for these non-local phenomena. However, this idea is not yet widely accepted because while there is evidence for quantum biology, quantum coherence in brain processing is so short-lived that it appears irrelevant to understanding consciousness. Neuroscience today says consciousness is generated by and localized in the brain because it emerges from brain activity. Alternatively, we propose that consciousness may not originate in the brain, although some aspects of human perception of consciousness may be dependent on the brain. We also suggest that awareness also extends beyond the brain. These non-physical, non-local properties of consciousness may be due to a non-local material effect, to consciousness being fundamental, or something else we have not yet discovered.

To begin an exploration of some of these non-physical theories, we present theoretical frameworks proposed by scientists from multiple disciplines, most of which include the idea that consciousness is fundamental, meaning that consciousness precedes the physical substrates (Chalmers, 1996; Currivan, 2017; Kastrup, 2017, 2021; Goff, 2019; Faggin, 2021a). Traditional materialists envision a world in which mathematics is more fundamental than physics, which is more fundamental than chemistry, which is, in turn, more fundamental than biology. Thus, in this way, physical processes are foundational to the generation of our biology. However, suppose we envision that consciousness is actually more foundational than physics. In that case, we can imagine that these other physical disciplines can arise from consciousness. In other words, if biology emerges from chemistry, chemistry from physics, and physics emerges from consciousness, then from this perspective, non-local consciousness phenomena would no longer be regarded as anomalous because consciousness can transcend some physical laws. Theories proposing this idea have been offered by Federico Faggin, Donald Hoffman, Bernardo Kastrup, Vernon Neppe, and numerous others. Most of these theories are speculative, while others are supported through mathematical arguments or empirical data (Hoffman et al., 2015; Neppe and Close, 2020; Faggin, 2021b). We briefly review a sample of non-local consciousness theories.

### Operational probabilistic theory

Federico Faggin starts with the assumption that reality emerges from the free-will communications of a vast number of conscious entities (Faggin, 2021a). Faggin calls the totality of what potentially and actually exists, *One.* Any self-knowing within this *one* is a transformation from potential existence into actual existence, where potential existence is the “reservoir” of self-knowing that has not yet manifested. Each new self-knowing brings rise to a *consciousness unit (CU).* The CU reflects the whole of *One* and is also part of *One* because *One* is never complete in its self-knowing process. Thus there must be continued self-knowing and continual generation of CUs, which explains an apparently growing number of conscious entities (Faggin, 2021a, p. 294). Faggin describes the CUs characteristics and how they combine into *self*, in which an entity with identity, awareness, and agency is dynamic, holistic, and self-knowing. Faggin views the physical world as a virtual reality metaphor, in which sophisticated avatars controlled by conscious beings interact with each other, where the body that controls the avatar exists outside the computer and is not part of the program. Similarly, the conscious entities that control physical bodies exist beyond the physical world that contains the body (Faggin, 2021b, p. 286).

### Interface theory of perception

Donald D. Hoffman proposes a model based on a mathematical structure called “conscious agents.” Space and time emerge from conscious agents’ exchanges (Hoffman, 2014). Hoffman proposes that our perceptions (i.e., the conscious agents) are not views of a grounded truth but are more like a personal computer’s operating system and interface (Hoffman, 2014, 2019). Perceptions allow us to interact dynamically with the world and survive and evolve in this environment but not be aware of its actual structure. Space-time and physical objects do not represent a universal objective reality but are species-specific components that provide an evolutionary advantage. Hoffman highlights that evolutionarily, perception of spacetime and the physical world are shaped by natural selection in such a way that obfuscates the truth that we are experiencing an interface rather than a universal objective reality and thus influences adaptive behaviors. He further claims that the equations of quantum mechanics can be derived from formalized descriptions of the interactions between conscious agents (Hoffman et al., 2015).

### Analytic idealism

Bernardo Kastrup proposes “analytic idealism” as a model for reality, in which the ground of existence is universal phenomenal consciousness (Kastrup, 2021). Analytic idealism is a metaphysics that postulates consciousness as Nature’s sole fundamental ground and that all natural phenomena are ultimately reducible to universal consciousness. He describes phenomenal consciousness as a raw subjective experience of awareness that differs from cognition, meta-cognition, self-awareness, or other higher mental functions. Meta-cognition allows humans to know that they are having an experience and also supports cognitive properties like reasoning and planning. Experiential consciousness or pure awareness can also occur without meta-cognition, as reported in classical mystical states. Because there is only one universal consciousness, individuated living beings are described as dissociated mental complexes of the “fundamentally unitary universal mind” (Kastrup, 2021, p. 267). This dissociation creates a subjective private inner world that can perceive itself as interacting with the transpersonal world. Matter in this model is described as the outward appearance of the inner experience as observed from across the dissociative boundary. Put another way,

*As experienced from the inside—that is, from the first-person perspective—each living being, plus the inanimate universe as a whole, is a conscious entity. But as experienced from outside—that is, from a[n illusory] second- or third-person perspective—our respective inner lives present themselves in the form of what we call matter, or physicality*…*all matter—is merely the name we give to what conscious inner life looks like from across its dissociative boundary. (Kastrup, 2021, pp. 267–268)*

### Triadic dimensional vortical paradigm

Vernon Neppe and Ed Close propose that the standard 4-dimensional model of physics (three dimensions of space and one of time) results in many contradictions or unexplained discrepancies (see Neppe and Close, 2020 for examples of apparent discrepancies). For example, using the Diophantine equation (a polynomial equation involving two or more unknowns and in which only integer solutions are allowed), Neppe states that the mass/energy of up-quarks and down-quarks produces an inequality that is unstable (Neppe and Close, 2015). To address these discrepancies, Neppe and Close describe a mathematical model in which we exist in a 9-dimensional finite, quantized, volumetric, spinning reality embedded in an infinite continuity (9D+). The model requires an extra component that they dub “gimmel,” which is mass and energy less. Close expresses that “gimmel is the connection between consciousness, life, and the atomic structure and that the potential for conscious life existed in the mutable mass and energy of quarks even before they became the first protium atoms of physical reality” (Close, 2018). The model proposes that the 4D world we ordinarily experience is the physical component of this 9D+ existence. Neppe and Close believe that the model has been empirically demonstrated with correspondences to normalized data for the mass-energy equivalence volumetric data for measured particles. They also claim that their model is mathematically valid at the micro, macro, and cosmological scales.

*Mathematically, gimmel necessarily has to exist in union with any particle in the universe for that particle to be stable. Without gimmel, the spinning (vortical) atoms would be unstable and asymmetrical about their axes and would, in effect, fly apart: Our world and the physical universe could not exist. (Neppe and Close, 2020, p. 4)*

### Zero-point field

Joachim Keppler (2018) proposes a theory where the energy of the vacuum is the basis for consciousness, the so-called “zero-point field” (Keppler, 2018). This is a theory of panpsychism where consciousness permeates the universe yet is only concentrated and apparent in certain circumstances. Unlike other panpsychism theories, it is not the “matter” that is conscious but empty space. However, the idea that matter is conscious may be incompatible with theoretical physics. If matter is conscious, particles may have the yet unknown property “consciousness.” Mathematically, particles are elementary because they cannot be assigned additional parameters than those currently assigned (e.g., a charge, spin, mass). Therefore, the idea that they are conscious is challenging to reconcile with physics. The zero-point field does not have the same problem. Keppler hypothesizes that the human brain is one of the physical mediums which can interact directly with the zero-point field by concentrating on it and thus experiencing consciousness. The details of this putative interaction are not currently known. However, the interesting element of this theory is that it leads to testable predictions, e.g., interactions between the brain (maybe through quantum phenomena as in the Orch OR theory), and the zero-point field could possibly be observed and measured. For example, there might be specific types of photon exchanges that would reveal this interaction.

### Orchestrated objective reduction theory

The Orch OR theory was developed by Stuart Hameroff and Sir Roger Penrose (Hameroff, 2021; Hameroff and Penrose, 2014). While the Copenhagen interpretation posits that the collapse of quantum states into a single state (the so-called “collapse of the wave function”) is determined by an observation (i.e., subjective reduction), Penrose’s objective reduction (OR) posits that it occurs when the energy difference (measured by spacetime curvature and mediated by gravity) of these states reaches an objective threshold (called the “Diósi–Penrose criterion”). Random proto-conscious moments of experience occur at each OR moment (Hameroff, 2021, p. 74). At the biological level, this OR would be orchestrated (Orch) by connective proteins (e.g., microtubule-associated proteins; MAPs) that influence this spacetime-separation of the qubits’ superimposed states. These quantum processes are performed by qubits formed on cellular microtubules by oscillating dipoles (the microtubule condensate), forming superposed resonance rings in helical pathways throughout the microtubule lattices. These oscillations are either electric or magnetic and are then amplified by neurons, leading to consciousness. This collective process corresponds to the orchestration of the objective reduction of quantum states in the brain (Orch-OR). The microtubules both influence and are influenced by the conventional synaptic activity of neurons. Hameroff later added that the condensates might travel across more considerable distances in the brain through dendritic-dendritic gap junctions (connections that allow much faster transfer of action potentials than synapses), generating gamma oscillations (high-frequency brain rhythms) associated with conscious perception, for example. This theory provides a straightforward mechanism that can be tested more easily than others. Experiments are underway to test the theory by evaluating if the proposed quantum interference is, in fact, present in microtubules and dampened by anesthesia (Kalra et al., 2020).

### Schooler hypothesis of subjective time

Psychologist Jonathan Schooler proposes subjective time as a new dimension of physics that would allow us to have a causal effect on the world (Schooler, 2014). This model proposes that one could conceive of the possibility of alternative dimensions of meta-perspective where each of us could move across time and raises the possibility that consciousness itself could have some causal role. In his model, a hierarchical cascade of conscious elements would have synchronization happening essentially like carrier waves. The lower level of waves has a particular rhythm. They are also synchronized, or cross-coupled, with the higher levels. In the same way that you can have very high-frequency waves or vibrations synced in with lower-frequency ones, in a sort of cross-coupling manner, you could also have the rhythms of the lower-level ones connected up to the higher-level ones. Through cross-frequency coupling, there potentially exists both top-down and bottom-up paths, explaining consciousness at a macroscopical level.

### Theory of double causality

Philippe Guillemant, a theoretical physicist, has proposed that trajectories between two spacetimes are not fixed within the block universe (Guillemant and Medale, 2019). The block universe is a model where the future is already realized and is implied by general relativity. Within this framework, Guillemant proposes a non-deterministic model of the block universe where consciousness and free will are mechanisms by which the exact path between two spacetime points is decided. He shows that this does not contradict the equations of physics. He also suggests that the irreversibility of time as we experience it might not be a fundamental property of the world but a statistical one. Statistically, time moves forward, but there might be rare instances where it could move backward. Similarly, he suggests that there might be future traces in the present. Although statistically, we will mostly see causal traces of the past, future traces may be experienced as observations of coordinated systems that past observations cannot explain. For example, one might observe an organized pattern that is not due to a specific causal effect in the past. In his model, he argues that the organization must come from the future as it has no causal past reason to exist. He states, “We can carry on doing physics, but we must be absolutely logical about it, considering our intentions as physical realities, with the added ingredient that they do not appear to depend solely on our brains but also on an information system outside spacetime” (Guillemant, 2016, p. 9).

### Summary of non-local consciousness models

Most of these theories assume that consciousness is fundamental and primary to all else. Our subjective intersection with this fundamental consciousness is described in different ways, such as being an interface, a dissociative boundary, or a consciousness unit. Moreover, the mechanistic structure of our world with consciousness as fundamental is explained in various ways (e.g., dimensions, conscious agents, *gimmel).*

However, it is important to note that physicalist theories still have a place in this framework. Even if consciousness is fundamental, these theories will inform on the mechanisms for the embodiment of consciousness into this materialistic reality (e.g., how the interface works). If we can perceive non-local information (as observed at the quantum scale), we likely still need to filter out the noise from the environment through uncertainty reduction, broadcast, and top-down processes for that information to become conscious. Predictive processes and updating the error prediction might be a crucial process to allow the perception of non-local information.

Another important point is that the IIT model could be a tool to study both physicalist and non-local theories of consciousness by including non-local properties into the spacetime postulates. In Section “Physicalist theories of consciousness,” we placed IIT as a physicalist theory of consciousness in the sense that it excludes non-local spacetime properties into the spacetime boundaries required for a physical system to be conscious, and all models are based on the conventional assumptions of spacetime. However, since IIT is only about information and systems, one may be able to test IIT for non-local consciousness. Spacetime properties could be included in the postulates (i.e., requirements for a physical substrate to be conscious) for the calculation of φ (e.g., quantum links between past and future) to see how this addition affects φ’s value. These non-local applications of ITT would allow for the non-local effects observed in quantum mechanics and the literature reviewed in Section “Phenomena suggested by a model of nonlocal consciousness.”

Just like physicalist theories need rigorous testing to validate them, non-local consciousness theories also need testing and validating. The key to fully validating a theory of consciousness (physicalist or non-local) is to make a prediction that can be experimentally tested and quantified, thus, validating or invalidating the prediction. Theories that cannot meet the prediction can be rejected or adjusted. Unfortunately, many theoretical predictions are challenging to test experimentally, and sometimes prediction confirmation might depend on future technological innovations. Often, the theory is built with abstract terms that need further precision and elaboration. The more precise the theory and the prediction, the more it lends itself to testing. Also, the theory may be demonstrated with mathematics and yet not currently be experimentally validated.

One very small step to explore the applicability of the concept of non-local consciousness models and the motivation for developing these models in the first place is driven by phenomena that are not accounted for by physicalist theories, as described in the next section. One reason that non-local consciousness models may be useful is that they allow for the possibility of the subjective experiences that are usually considered impossible by physicalist models or simply ignored because of the basic assumptions on which they are built.

## Phenomena suggested by a model of non-local consciousness

In the next section, we propose specific phenomena that we would expect to see if non-local consciousness theories are correct.

### Phenomenon #1: Perceiving information about distant locations

If consciousness were non-local, then an individual ought to be able to perceive information beyond the reach of the brain, body, and senses. For example, one might be able to gain information about a person, place, or object at a distant location. Such abilities are described as part of a classified US government program that ran from 1972 to 1995, which sought to use non-local consciousness for espionage (May and Marwaha, 2018). That program conducted over 500 operational missions, some of which are said to have resulted in actionable intelligence and also several hundred controlled experimental trials. The latter was evaluated by a professor of statistics and skeptical psychology professional. Both concluded that the evidence in those studies was statistically significant and could not be attributed to methodological flaws (Mumford et al., 1995; Utts, 2016). In a typical experimental session, a “viewer” would enter a relaxed state. An interviewer would give them a random number designating the desired target and then ask them to describe and/or draw any information they perceived about that target. Both viewer and interviewer were blind to the target. Multiple meta-analyses of public domain and declassified experiments of this type have been conducted, and the results showed highly positive evidence in favor of a genuine phenomenon (Milton, 1997; Dunne and Jahn, 2003; Baptista et al., 2015; Cardeña, 2018). This apparent ability is now used for other practical applications, such as predicting stock market movements (Harary and Targ, 1985; Kolodziejzyk, 2013; Smith et al., 2014), locating missing persons (Mcmoneagle and May, 2004), and finding previously unknown archaeological sites (Schwartz, 2005, 2019).

### Phenomenon #2: Perceiving information from another person

If consciousness were non-local, an individual might be able to receive information about another, isolated person’s mental activity from a distance. Numerous well-controlled laboratory studies have observed this apparent phenomenon using the *ganzfeld* protocol, one of the most-repeated non-local consciousness studies. Ganzfeld originates from a German word meaning “whole field,” and Gestalt psychologists initially developed the protocol. First, a person is exposed to low-level, unpatterned sensory stimuli (e.g., red light diffused to the eyes and white noise played through headphones). Meanwhile, a second, isolated person attempts to mentally “send” a target image randomly selected out of a pool of four possible images, which was randomly selected out of a database of many such pools. The chance of the “receiving” person correctly selecting the actual image is thus 25%. Over 120 published experiments have used this protocol, comprising about 4,000 individual trials, and the overall hit rate was just over 30%. Multiple reviews and meta-analyses on this protocol have also been conducted (Storm et al., 2010; Baptista et al., 2015; Cardeña, 2018; Storm and Tressoldi, 2020). These results have been discussed and debated in one of the principal journals in academic psychology, *Psychological Bulletin* (Bem and Honorton, 1994; Hyman, 2010; Storm et al., 2010).

In a conceptually similar design, rather than testing whether one person could select a correct image sent by another isolated person, the person’s unconscious physiological state was intentionally influenced by a second person who was asked to focus their attention on them. These studies have typically used measures such as electrodermal activity (Braud and Schlitz, 1983; Radin et al., 2008), electroencephalography (EEG) activity (Standish et al., 2004; Richards et al., 2005), and functional magnetic resonance imaging (Standish et al., 2003; Achterberg et al., 2005). To date, there have been three meta-analyses for this class of studies, with each reporting statistically significant outcomes (Schmidt et al., 2004; Schmidt, 2012, 2015). Using this experimental paradigm, researchers discovered that the prior beliefs of the investigators were an important element in the observed outcomes. That is, working with the same subject populations, protocol, equipment, and analyses, skeptical investigators obtained null results, but investigators more open to the possibility of an effect obtained significant results (Watt et al., 2002; Schlitz et al., 2006). These investigator-specific effects have been documented in psychology and are called “experimenter effects” (Palmer and Millar, 2015). Thus, it is challenging to ascertain if results are solely influenced by the experimenter effect (i.e., intentions of the investigator) or if there are intrinsic effects. Multiple-experimenter studies have been posed as a solution to solving this issue in psychological studies (Bierman and Jolij, 2020).

### Phenomenon #3: Perceiving the future

If consciousness were non-local, one might be able to perceive information from non-inferable future events. Experiments testing this idea have shown that people’s physiology has reacted to randomly selected future events (Radin and Pierce, 2015), including electrodermal (Radin, 1997) and electrocortical activity (Radin and Lobach, 2007; Radin and Borges, 2009; Radin et al., 2011), and heart rate (McCraty et al., 2004; Tressoldi et al., 2009). These laboratory studies apparently demonstrate that the body can react to randomly selected stimuli approximately 1–10 s in the future. Erotic and negative images produce more robust responses than emotionally neutral pictures, and pre-responses generally manifest in the same direction as the body would typically respond after exposure to a stimulus. Meta-analyses have evaluated multiple laboratory studies with positive effect sizes (Mossbridge et al., 2012, 2014; Storm et al., 2012; Mossbridge and Radin, 2018; Honorton et al., 2018). For example, Mossbridge et al. (2012) analyzed 26 studies where unpredictable stimuli were presented and physiological activity was collected before, during, and after the stimuli. There was a pre-stimulus effect demonstrating a physiological response prior to the unpredictable stimuli (fixed effect: overall effect size = 0.21, 95% CI = 0.15 – 0.27, *z* = 6.9, *p* < 2.71 × 10^–12^; Mossbridge et al., 2012).

Implicit bias tests with a retrocausal element provide similar findings. In one paradigm, a classic perceptual priming task was reversed, such that the prime occurred after the target images. For example, in one task, the prime “happy” might typically occur prior to the target picture of a flower. In a reverse priming task, the flower image would appear *before* the prime “happy.” These reverse priming tasks found slower response times when the prime/target pairs were incongruent (sad/flower) versus congruent (happy/flower), just as the classic task would, even though the prime occurred *prior* to the target. Some 90 independent replications of these experiments have provided evidence for a highly significant effect (overall effect size = 0.09, *z* = 6.4, *p* = 1.2 × 10^–10^; Bem et al., 2015).

### Phenomenon #4: Apparent cognitive abilities beyond the experience/learning/skill of the person exhibiting them

If consciousness were non-local, then people might be able to gain cognitive skills without previous experience or training in those skills.

An example is the phenomenon of a person speaking a language unknown to, or xenoglossy. This phenomenon has been reported since ancient times. It refers to the ability of an individual to speak or write a language that they presumably did not know and could not have acquired by ordinary means. For example, in 400 BC, Plato mentions priestesses on the Island of Delos who spoke “in tongues.” There are also descriptions in the Bible (Corinthians 14:1-40 and Acts 2:4).

Another example is Indriði Indriðason (1883–1912), who apparently spoke multiple languages he did not know (Haraldsson, 2012). Similarly, Alec Harris spoke at length to witness Sir Alexander Cannon in Hindustani and Tibetan, two languages that Harris would have had no way of knowing, but Sir Alexander did know (Vandersande, 2008, p. 113). Other xenoglossy cases have also been documented by University of Virginia scientist Ian Stevenson (Stevenson and Pasricha, 1979, 1980). While anecdotal and subject to the known biases of experiential reports, these cases have been meticulously well-documented. Similar cases of “acquired” and “spontaneous savants” refer to individuals who, either through a traumatic event or with no apparent cause at all, suddenly gain exceptional musical or mathematical skills (Treffert, 2009).

### Phenomenon #5: Non-local consciousness experiences are common

If consciousness were non-local, such experiences would be highly prevalent in all humans. And indeed, non-local experiences can be found throughout history, across all cultures, and at all educational levels. Formal prevalence studies have been conducted for almost 50 years, with rates ranging from 10% to 97%, depending on the population surveyed (Bourguignon, 1976; Palmer, 1979; Haraldsson, 1985, 2011; Greeley, 1987; Haraldsson and Houtkooper, 1991; Ross and Joshi, 1992; McClenon, 1993; Cohn, 1994; Castro et al., 2014; Wahbeh et al., 2018). Another survey of the general public, scientists, and engineers in the United States found that over 90% had experienced at least one of 25 of these experiences (Wahbeh et al., 2018). With prevalence rates being well-above 10% of most populations surveyed, it is evident that these phenomena, at least in their subjective reports, are more frequent than commonly supposed.

### Phenomenon #6: Cognitive abilities can be retained when the brain is seriously compromised

We usually assume that the brain is the body’s driver, and if the brain is not working well, the body should not work. Suppose this is wrong and consciousness is not entirely dependent on the physical function of the brain. In that case, cognition, perception, and memory may continue to operate normally even when the brain would not be considered functional. This is consistent with what we see in a phenomenon called *terminal lucidity*. Terminal lucidity is a label given to a phenomenon in which patients with terminal neurodegenerative conditions display apparently normal cognitive function and mental clarity during the period preceding death (hours to days). While such experiences would seem impossible based on known principles of neuroscience and neuroanatomy, they have been reported in the medical literature for over 250 years (Nahm et al., 2012).

Terminal lucidity, also called paradoxical lucidity, has occurred in conditions such as waking from a long-term coma, dementia due to advanced Alzheimer’s disease, brain abscesses, tumors, strokes, and meningitis (Nahm et al., 2012). A recent study of terminal lucidity reviewed 124 cases in dementia patients and found that in “more than 80% of these cases, complete remission with the return of memory, orientation, and responsive verbal ability was reported by observers of the lucid episode” (Batthyány and Greyson, 2021). For example, one reported case involved a patient with cancer that had metastasized to the brain, with little functional brain tissue remaining. However, an hour before the patient died, he regained awareness and conversed with his family for about 5 min before passing away (Nahm et al., 2012). Most terminal lucidity cases are retrospective case reports (Kelly et al., 2007; Nahm and Greyson, 2009; Nahm et al., 2012; Mashour et al., 2019; Batthyány and Greyson, 2021), but a few are prospective. Macleod and colleagues prospectively observed terminal lucidity cases (Macleod, 2009), as did Fenwick and colleagues (Fenwick et al., 2010). In these cases, the patients demonstrated normal cognitive abilities just prior to death, contrary to what objective medical findings would have predicted (e.g., EEG, neuroimaging). These patients are operating in an anomalous manner that brings into question the idea that the body is a “puppet” controlled from the inside (the brain) and that perhaps it can function alternately in some instances. Perhaps there are aspects of consciousness that could be “outside” of the body controlling it. The lucid mental functioning associated with these patients’ behavior is challenging to explain under the assumption that one’s sense of identity, memory, and awareness solely depends on brain activity.

### Summary

In sum, we presented six phenomena regarding aspects related to non-local consciousness. Reports of individuals perceiving information from distant locations, from another person, from the future, where people gain skills beyond their normal capacity, or when the brain is apparently non-functional, have been documented in anecdotal and experimental contexts. In addition, these phenomena are ubiquitous worldwide. Note that these examples are not meant to provide definitive evidence for non-local consciousness, nor provide a comprehensive list of such phenomena, but rather to highlight that certain commonly reported phenomena, and some rare effects, present clear challenges to prevailing physicalist models of consciousness.

Of course, given the significant theoretical importance of these phenomena, each example supporting these predictions has evoked critical responses. The critiques have tended to fall into two classes. First, the phenomena suggested by these examples are deemed impossible because they violate the basic limiting principles of science. Therefore, the only possible way to interpret experiments reporting positive results is that they most likely involve flaws, fraud, or both. Critical reactions to anecdotal reports have also tended to focus on their subjective nature and the many ways that such experiences can be misinterpreted as illusions, misperceptions, or distorted memories. Such critiques can be answered by pointing out that some of the anecdotal reports involved hundreds to thousands of documented case studies, and all the experiments mentioned involved controlled experimental paradigms that were repeated in multiple laboratories and dozens to over a hundred independent replications, with overall highly significant meta-analytic outcomes (Cardeña, 2018). In some of the earliest experiments, methodological flaws were discovered but later corrected with similar results, so insisting that flaws or fraud can be the only possible explanations is not supported by analysis of the data.

The second category of critique is that perhaps the results could be accounted for by one or more physicalist explanations that we do not understand yet, given the state of the science. For example, perhaps some material explanation will eventually arise for how someone with severe brain atrophy and neurofibrillary tangles, or who was in a deep coma for an extended period, could nevertheless suddenly become lucid and maintain a coherent conversation with loved ones shortly before death. Alternatively, perhaps if it is established that the brain has quantum biological properties, then that might provide a plausible substrate for perceptual non-locality. That is, a brain that is partially acting in a quantum manner could possibly account for all these anomalous phenomena. A quantum brain would have non-local properties, so our sensory system would be spread out in space and time, and it might also have observational properties. However, even if this was true, it would not tell us anything about the nature or source of our subjective awareness. That is, from the quantum brain perspective, these phenomena would be completely explained as a purely physical phenomena (albeit within the context of the not-quite-physical nature of the quantum world).

## The scientific process and perspective in the face of a paradigm shift

Our call to test non-local consciousness theories is not a proposal to discard physicalist theories. There is no question that, as a set of assumptions, materialism has proven to be outstandingly successful in elucidating the nature of physical reality, and it will likely continue to be useful. However, the phenomena we have highlighted here bring some level of doubt to the ability of physicalist theories to explain *everything*, including the nature, origin, and capacities of consciousness. Here we propose that materialism be viewed as a special case of a more comprehensive metaphysic, one that includes consciousness in some fundamental way. This approach is akin to regarding classical physics as a special case, one that describes a limited domain of the physical world. Quantum mechanics, too, is probably a special case because, so far, it is not compatible with relativistic physics. These “modern” physical theories are more comprehensive than classical physics and are special cases.

Promoting the value of more comprehensive models of reality can be challenging. As Max Planck said, “A new scientific truth does not triumph by convincing its opponents and making them see the light, but rather because its opponents eventually die, and a new generation grows up that is familiar with it” (Planck, 1950, p. 33). Researchers testing if this statement was true found that, indeed, acceptable scientific models and concepts became more varied after leaders in their field died (Azoulay et al., 2019).

A classic example of shifts in worldviews in science is the case of black holes. Imagine it is 1921, and we asked, “Do black holes exist?” In 1915, Karl Schwarzschild solved Einstein’s equations of general relativity for the limited case of a single spherical non-rotating mass. In the process, he discovered the possibility that under extreme gravitational conditions, space could collapse upon itself. Einstein denied that these “black holes” could possibly form. In 1939, he published a paper arguing that a star collapsing would spin faster and faster, eventually spinning at the speed of light with infinite energy, well before the point that it would collapse into a singularity. It was not until the 1960s when Roger Penrose published more detailed models showing how black holes could form, that other physicists considered them viable. A half-century later, astronomers finally observed a black hole (The Event Horizon Telescope Collaboration). In fact, a team of Harvard scientists just released an image of Sagittarius A-star, a black hole at the center of our Milky Way galaxy (McDermott-Murphy, 2022).

We propose that today’s understanding of the evidence for non-local consciousness is similar to what was understood about black holes in 1921. A jury of leading scientists in 1921 charged with deciding if black holes existed would have weighed the pros and cons of existing theory and data, they would have consulted with Einstein, and they would have almost certainly decided that black holes could not exist and therefore did not exist. As we know today, they would have been wrong.

## Conclusion

Intriguing phenomena are alluding to consciousness being associated with – but not limited by – brain activity. We are in a position similar to those who were studying the possibility of black holes a century ago. Perhaps in 50 years, we will look back on the current transitional period between materialistic and post-materialistic paradigms in science and more clearly understand why we could not have possibly grasped the whole picture.

We can learn from the black hole example to release our desire to prove non-local consciousness and instead remain in a state of curiosity, focusing on methods and improved measures. Even if it were not possible to definitively demonstrate that consciousness is non-local but in the process of determining that, it was discovered that there were non-local aspects of consciousness that we learned more about and controlled to some extent, our world would be radically transformed with the shift in the understanding of our capacities and its practical applications. The systematic scientific study of consciousness is still in its infancy, and thus, we are at the very beginning of understanding the right questions to ask.

This review also calls for humility, open-mindedness, and collaboration in science. Is it possible to remain neutral about the various theories of consciousness? Perhaps physicalist theories will be tested and shown to be relevant in particular situations. Perhaps non-materialist theories will also be tested and shown to be valid in other situations. Could it be that multiple theories of consciousness are tested and found viable? If so, what would that mean about the nature of reality? Can these theories be evaluated for similarities and differences, perhaps combining some and ultimately testing them? Templeton World Charity Foundation Program ‘Accelerating Research on Consciousness’ has spearheaded such an initiative for physicalist theories. The same could be implemented for non-physicalist theories (Templeton World Charity Foundation, 2022). Is there an interaction between a non-local consciousness interfacing with the physical and/or quantum brain that is persuasively describable? Remaining open and flexible about these possibilities is essential in supporting the birth of new ideas. Remaining humble allows us to review other theories without prejudice.

To further assess the vast number of theories of consciousness, physicalist and non-local, we invite theorists to attempt to make their theories increasingly precise so that abstract terms become quantifiable predictions that can be confirmed or refuted. Furthermore, theorists could attempt to use similar language/terms to improve the clarity regarding the distinctions and commonalities across theories. Criteria could be developed, allowing researchers to easily determine the nature/aspect of consciousness discussed by the theory, the proposed processes that explain how non-local consciousness may interact with physical substrates, and the precise predictions to validate it. Physicalist theories might be closer to validating or invalidating their predictions if the assumption about the nature of consciousness being generated from the brain is correct. However, these predictions may never address the possibility that consciousness is a fundamental property of reality with non-local properties (i.e., they address physical mechanisms but not the nature of consciousness itself).

In conclusion, our reported phenomena of non-local consciousness present intriguing examples that should be addressed when evaluating whether consciousness may be more than an emergent property of brain activity. Despite sophisticated physicalist theories of consciousness dependent on brain function, these examples apparently demonstrate non-local aspects of consciousness, perceiving information in a way that is not limited by our conventional understanding of time and space and that is not dependent on the brain function. Many of these data have been observed with objective measures in the laboratory in a valid and reliable way or collected in the field with impeccable methods and exclusion of fraud. While materialism explains much in our world, it does not explain everything, including these phenomena. Non-materialist theories encompassing consciousness as fundamental and/or non-local may provide a pathway to understanding these phenomena. Perhaps holding the hypothetical assumption that consciousness *is* fundamental and focusing on what we can learn about the mechanism, mediators, moderators, and practical applications of non-local consciousness will reveal novel areas to explore.

## Author contributions

HW contributed to the conceptualization, funding acquisition, project administration, and writing – original draft preparation, review, and editing. DR and AD contributed to the conceptualization, funding acquisition, and writing – original draft preparation, review, and editing. CC contributed to the conceptualization and writing – review and editing. All authors contributed to the article and approved the submitted version.

## References

[B1] Achterberg J. Cooke K. Richards T. Standish L. J. Kozak L. Lake J. (2005). Evidence for correlations between distant intentionality and brain function in recipients: A functional magnetic resonance imaging analysis. *J. Altern. Complement. Med.* 11 965–971. 10.1089/acm.2005.11.965 16398587

[B2] Albantakis L. (2020). “Integrated information theory,” in *Beyond neural correlates of consciousness*, eds Kirkeby-Hinrup A. Mogensen J. Overgaard M. (Abingdon: Routledge).

[B3] Albantakis L. Hintze A. Koch C. Adami C. Tononi G. (2014). Evolution of integrated causal structures in animats exposed to environments of increasing complexity. *PLoS Comput. Biol.* 10:e1003966. 10.1371/journal.pcbi.1003966 25521484 PMC4270440

[B4] Aru J. Bachmann T. Singer W. Melloni L. (2012). Distilling the neural correlates of consciousness. *Neurosci. Biobehav. Rev.* 36 737–746. 10.1016/j.neubiorev.2011.12.003 22192881

[B5] Azoulay P. Fons-Rosen C. Zivin J. S. G. (2019). Does science advance one funeral at a time? *Ame. Econ. Rev.* 109 2889–2920. 10.1257/aer.20161574 31656315 PMC6814193

[B6] Baars B. J. (2005). Global work-space theory of consciousness: Toward a cognitive neuroscience of human experience. *Prog. Brain Res.* 150 45–53. 10.1016/S0079-6123(05)50004-9 16186014

[B7] Baars B. J. Geld N. Kozma R. (2021). Global Workspace Theory (GWT) and prefrontal cortex: Recent developments. *Front. Psychol.* 12:749868. 10.3389/fpsyg.2021.749868 34899489 PMC8660103

[B8] Baptista J. Derakhshani M. Tressoldi P. E. (2015). “Explicit anomalous cognition: A review of the best evidence in ganzfeld, forced choice, remote viewing and dream studies,” in *Parapsychology: A handbook for the 21st century*, eds Cardeña E. Palmer J. Marcusson-Clavertz D. (Jefferson, NC: McFarland & Company, Inc., Publishers), 192–214.

[B9] Barrett L. F. (2016). The theory of constructed emotion: An active inference account of interoception and categorization. *Soc. Cogn. Affect. Neurosci.* nsw154. 10.1093/scan/nsw154 27798257 PMC5390700

[B10] Batthyány A. Greyson B. (2021). Spontaneous remission of dementia before death: Results from a study on paradoxical lucidity. *Psychol. Conscious.* 8 1–8. 10.1037/cns0000259

[B11] Bem D. J. Honorton C. (1994). Does psi exist? Replicable evidence for an anomalous process of information transfer. *Psychol. Bull.* 115 4–18. 10.1037/0033-2909.115.1.4

[B12] Bem D. J. Tressoldi P. Rabeyron T. Duggan M. (2015). Feeling the future: A meta-analysis of 90 experiments on the anomalous anticipation of random future events. *F1000Res.* 4:1188. 10.12688/f1000research.7177.2 26834996 PMC4706048

[B13] Bierman D. Jolij J. J. (2020). Dealing with the experimenter effect. *J. Sci. Explor.* 34 703–709. 10.31275/20201871

[B14] Blumenfeld H. Taylor J. (2003). Why do seizures cause loss of consciousness? *Neuroscientist* 9 301–310. 10.1177/1073858403255624 14580115

[B15] Bourguignon E. (1976). *Possession.* Novato,CA: Chandler & Sharp Publishers.

[B16] Braud W. Schlitz M. (1983). Psychokinetic influence on electrodermal activity. *J. Parapsychol.* 47 95–119.

[B17] Cardeña E. (2018). The experimental evidence for parapsychological phenomena: A review. *Am. Psychol.* 73 663–677. 10.1037/amp0000236 29792448

[B18] Castro M. Burrows R. Wooffitt R. (2014). The paranormal is (still) normal: The sociological implications of a survey of paranormal experiences in Great Britain. *Sociol. Res. Online* 19:16. 10.5153/sro.3355

[B19] Chalmers D. J. (1996). *The conscious mind: In search of a fundamental theory.* Oxford: Oxford University Press.

[B20] Clark A. (2013). Whatever next? Predictive brains, situated agents, and the future of cognitive science. *Behav. Brain Sci.* 36 181–204. 10.1017/S0140525X12000477 23663408

[B21] Cleeremans A. Achoui D. Beauny A. Keuninckx L. Martin J.-R. Muñoz-Moldes S. (2020). Learning to Be Conscious. *Trends Cogn. Sci.* 24 112–123. 10.1016/j.tics.2019.11.011 31892458

[B22] Close E. R. (2018). *Gimmel, life and consciousness.* Available online at: http://www.erclosetphysics.com/2018/05/gimmel-life-and-consciousness.htmlAD (accessed May 27, 2022).

[B23] Cohn S. A. (1994). A survey on Scottish second sight. *J. Soc. Psych. Res.* 59 385–400. 10.1001/jamaoncol.2021.6987 34967848 PMC8719276

[B24] Currivan J. (2017). *The cosmic hologram: In-formation at the center of creation.* New York, NY: Simon and Schuster.

[B25] de Graaf T. A. Hsieh P.-J. Sack A. T. (2012). The “correlates” in neural correlates of consciousness. *Neurosci. Biobehav. Rev.* 36 191–197. 10.1016/j.neubiorev.2011.05.012 21651927

[B26] Dehaene S. Charles L. King J.-R. Marti S. (2014). Toward a computational theory of conscious processing. *Curr. Opin. Neurobiol.* 25, 76–84. 24709604 10.1016/j.conb.2013.12.005 PMC5635963

[B27] Dehaene S. Naccache L. Cohen L. Bihan D. L. Mangin J.-F. Poline J.-B. (2001). Cerebral mechanisms of word masking and unconscious repetition priming. *Nat. Neurosci.* 4 752–758. 10.1038/89551 11426233

[B28] Dehaene S. Sergent C. Changeux J.-P. (2003). A neuronal network model linking subjective reports and objective physiological data during conscious perception. *Proc. Natl. Acad. Sci. U.S.A.* 100, 8520–8525. 12829797 10.1073/pnas.1332574100 PMC166261

[B29] Dossey L. (1994). Healing and the mind: Is there a dark side? *J. Sci. Explor.* 8 73–90.

[B30] Dunne B. J. Jahn R. G. (2003). Information and uncertainty in remote perception research. *J. Sci. Explor.* 17 207–241. 10.1016/j.explore.2007.03.010 17560347

[B31] Faggin F. (2021a). “Consciousness Comes First,” in *Consciousness unbound: Liberating mind from the tyranny of materialism*, eds Kelly E. F. Marshall P. (Lanham, MD: Rowman & Littlefield Publishers), 283–322.

[B32] Faggin F. (2021b). *Silicon: From the invention of the microprocessor to the new science of consciousness.* Sea, CA: Waterside Productions.

[B33] Fenwick P. Lovelace H. Brayne S. (2010). Comfort for the dying: Five year retrospective and one year prospective studies of end of life experiences. *Arch. Gerontol. Geriatr.* 51 173–179. 19913927 10.1016/j.archger.2009.10.004

[B34] Friston K. (2010). The free-energy principle: A unified brain theory? *Nat. Rev. Neurosci.* 11 127–138. 10.1038/nrn2787 20068583

[B35] Gershman S. J. (2019). The generative adversarial brain. *Front. Artif. Intell.* 2:18. 10.3389/frai.2019.00018 33733107 PMC7861215

[B36] Goff P. (2019). *Galileo’s error: Foundations for a new science of consciousness.* New York, NY:: Pantheon Books.

[B37] Greeley A. (1987). Mysticism goes mainstream. *Am. Health* 7 47–49.

[B38] Grossberg S. (2013a). Adaptive resonance theory. *Scholarpedia* 8:1569. 10.4249/scholarpedia.1569

[B39] Grossberg S. (2013b). Adaptive resonance theory: How a brain learns to consciously attend, learn, and recognize a changing world. *Neural Netw.* 37 1–47. 10.1016/j.neunet.2012.09.017 23149242

[B40] Guillemant P. (2016). *Theory of double causality. Time matters review.* Available online at: http://www.guillemant.net/english/Theory_of_Double_Causality.pdf

[B41] Guillemant P. Medale M. (2019). A unique but flexible space–time could challenge multiverse theories. *Ann. Phys.* 409:167907. 10.1016/j.aop.2019.05.005

[B42] Hameroff S. (2021). ‘Orch OR’ is the most complete, and most easily falsifiable theory of consciousness. *Cogn. Neurosci.* 12 74–76. 10.1080/17588928.2020.1839037 33232193

[B43] Hameroff S. Penrose R. (2014). Consciousness in the universe: A review of the ‘Orch OR’ theory. *Phys. Life Rev.* 11 39–78. 10.1016/j.plrev.2013.08.002 24070914

[B44] Haraldsson E. (1985). Representative national surveys of psychic phenomena: Iceland, Great Britain, Sweden, USA and Gallup’s multinational survey. *J. Soc. Psych. Res.* 53 145–158.

[B45] Haraldsson E. (2011). Psychic experiences a third of a century apart: Two representative surveys in Iceland with an international comparison. *J. Soc. Psych. Res.* 75:76.

[B46] Haraldsson E. (2012). Further facets of Indridi indridason’s mediumship, including “transcendental” music, direct speech, xenoglossy and light phenomena. *J. Soc. Psych. Res.* 76 129–149.

[B47] Haraldsson E. Houtkooper J. M. (1991). Psychic experiences in the multinational human values study: Who reports them. *J. Am. Soc. Psych. Res.* 85 145–165.

[B48] Harary K. Targ R. (1985). A new approach to forecasting commodity futures. *Psi Res.* 4 79–88.

[B49] Haun A. Tononi G. (2019). Why does space feel the way it does? Towards a principled account of spatial experience. *Entropy* 21:1160. 10.3390/e21121160

[B50] Hebb D. O. Penfield W. (1940). Human behavior after extensive bilateral removal from the frontal lobes. *Arch. Neurol. Psychiatry* 44 421–438. 10.1001/archneurpsyc.1940.02280080181011

[B51] Hoffman D. D. (2014). The origin of time in conscious agents. *Cosmology* 18 494–520.

[B52] Hoffman D. D. (2019). *The case against reality: Why evolution hid the truth from our eyes.* New York, NY: W.W. Norton & Company.

[B53] Hoffman D. D. Singh M. Prakash C. (2015). The interface theory of perception. *Psychon Bull. Rev.* 22 1480–1506. 10.3758/s13423-015-0890-8 26384988

[B54] Hohwy J. (2013). *The predictive mind.* Oxford: Oxford University Press. 10.1093/acprof:oso/9780199682737.001.0001

[B55] Hohwy J. Seth A. (2020). Predictive processing as a systematic basis for identifying the neural correlates of consciousnes. *Psyarxiv* [Preprint]. 10.33735/phimisci.2020.II.64

[B56] Honorton C. Ferrari D. B. Hansen G. (2018). “Meta-analysis of Forced-Choice Precognition Experiments (1935–1987),” in *The star gate archives: Reports of the united states government sponsored psi program, 1972-1995. volume 2: Remote viewing, 1985-1995*, eds May E. C. Marwaha S. B. (Jefferson, NC: McFarland & Company, Inc., Publishers), 291.

[B57] Huang Y. Rao R. P. N. (2011). Predictive coding. *Wiley Interdiscip. Rev. Cogn. Sci.* 2 580–593. 10.1002/wcs.142 26302308

[B58] Hudetz A. G. (2012). General anesthesia and human brain connectivity. *Brain Connect.* 2 291–302. 10.1089/brain.2012.0107 23153273 PMC3621592

[B59] Hyman R. (2010). Meta-analysis that conceals more than it reveals: Comment on storm et al. (2010). *Psychol. Bull.* 136 486–490. 10.1037/a0019676 20565165

[B60] Kalra A. P. Hameroff S. Tuszynski J. Dogariu A. Sachin N. Gross P. J. (2020). *Anesthetic gas effects on quantum vibrations in microtubules – Testing the Orch OR theory of consciousness. OSF.* Available online at: https://osf.io/zqnjd/ (accessed May 5, 2022).

[B61] Kastrup B. (2017). Self-transcendence correlates with brain function impairment. *J. Cogn. Neuroethics* 4 33–42.

[B62] Kastrup B. (2021). “Analytic idealism and psi: How a more teneable metaphysics neutralizes a physicalist taboo,” in *Consciousness unbound: Liberating mind from the tyranny of materialism*, eds Kelly E. F. Marshall P. (Lanham: Rowman & Littlefield Publishers), 257–283.

[B63] Kelly E. W. Greyson B. Kelly E. F. (2007). “Unusual experiences near death and related phenomena,” in *Irreducible mind: Toward a psychology for the 21st century*, eds Kelly E. F. Kelly E. W. Crabtree A. Gauld A. Grosso M. Greyson B. (Lanham: Rowman and Littlefield), 367–421.

[B64] Keppler J. (2018). The role of the brain in conscious processes: A new way of looking at the neural correlates of consciousness. *Front. Psychol.* 9:1346. 10.3389/fpsyg.2018.01346 30123156 PMC6085561

[B65] Koch C. (2018). What is consciousness? *Nature* 557 S8–S12. 10.1038/d41586-018-05097-x 29743705

[B66] Koch C. Massimini M. Boly M. Tononi G. (2016). Neural correlates of consciousness: Progress and problems. *Nat. Rev. Neurosci.* 17, 307–321. 10.1038/nrn.2016.22 27094080

[B67] Kolodziejzyk G. (2013). Greg Kolodziejzyk’s 13-year associative remote viewing experiment results. *J. Parapsychol.* 76 349–368.

[B68] Lamme V. A. F. (2006). Towards a true neural stance on consciousness. *Trends Cogn. Sci.* 10 494–501. 10.1016/j.tics.2006.09.001 16997611

[B69] Lamme V. A. F. (2010). How neuroscience will change our view on consciousness. *Cogn. Neurosci.* 1 204–220. 10.1080/17588921003731586 24168336

[B70] Lamme V. A. Roelfsema P. R. (2000). The distinct modes of vision offered by feedforward and recurrent processing. *Trends Neurosci.* 23 571–579. 10.1016/s0166-2236(00)01657-x 11074267

[B71] Lau H. (2019). Consciousness, metacognition, & perceptual reality monitoring. *PsyArXiv* [Preprint]. 10.31234/osf.io/ckbyf

[B72] Lau H. Rosenthal D. (2011). Empirical support for higher-order theories of conscious awareness. *Trends Cogn. Sci.* 15 365–373. 10.1016/j.tics.2011.05.009 21737339

[B73] Leopold D. A. Logothetis N. K. (1999). Multistable phenomena: Changing views in perception. *Trends Cogn. Sci.* 3 254–264. 10.1016/S1364-6613(99)01332-7 10377540

[B74] Macleod A. D. S. (2009). Lightening up before death. *Palliat. Support. Care* 7 513–516. 10.1017/S1478951509990526 19939314

[B75] Mashour G. A. Frank L. Batthyany A. Kolanowski A. M. Nahm M. Schulman-Green D. (2019). Paradoxical lucidity: A potential paradigm shift for the neurobiology and treatment of severe dementias. *Alzheimers Dement. J. Alzheimers Assoc.* 15 1107–1114. 10.1016/j.jalz.2019.04.002 31229433

[B76] Mashour G. A. Roelfsema P. Changeux J.-P. Dehaene S. (2020). Conscious processing and the global neuronal workspace hypothesis. *Neuron* 105, 776–798. 10.1016/j.neuron.2020.01.026 32135090 PMC8770991

[B77] May E. C. Marwaha S. B. (2018). *The star gate archives: reports of the united states government sponsored psi program, 1972-1995*, Vol. 1. Jefferson, NC: McFarland & Company, Inc., Publishers.

[B78] McClenon J. (1993). Surveys of anomalous experience in Chinese. Japanese, and American samples. *Sociol. Relig.* 54 295–302.

[B79] McCraty R. Atkinson M. Bradley R. T. (2004). Electrophysiological evidence of intuition: Part 1. The surprising role of the heart. *J. Altern. Complement. Med.* 10 133–143. 10.1089/107555304322849057 15025887

[B80] McDermott-Murphy C. (2022). *The dawn of a new era in astronomy. Harvard Gazette.* Available online at: https://news.harvard.edu/gazette/story/2022/05/the-dawn-of-a-new-era-in-astronomy/

[B81] Mcmoneagle J. W. May E. C. (2004). “The possible role of intention, attention and expectation in remote viewing,” in *Proceedings of Presented Papers. The Parapsychological Association 47th Annual Convention*, (Vienna: Vienna University).

[B82] Milton J. (1997). Meta-analysis of free-response ESP studies without altered states of consciousness. *J. Parapsychol.* 61 279–319.

[B83] Mossbridge J. Radin D. (2018). Precognition as a form of prospection: A review of the evidence. *Psychol. Conscious.* 5:78.

[B84] Mossbridge J. Tressoldi P. Utts J. (2012). Predictive physiological anticipation preceding seemingly unpredictable stimuli: A meta-analysis. *Front. Psychol.* 3:390. 10.3389/fpsyg.2012.00390 23109927 PMC3478568

[B85] Mossbridge J. Tressoldi P. Utts J. Ives J. A. Radin D. Jonas W. B. (2014). Predicting the unpredictable: Critical analysis and practical implications of predictive anticipatory activity. *Front. Hum. Neurosci.* 8:146. 10.3389/fnhum.2014.00146 24723870 PMC3971164

[B86] Mumford M. Rose A. Goslin D. (1995). *An evaluation of remote viewing: Research and applications.* Arlington: American Institutes for Research.

[B87] Nahm M. Greyson B. (2009). Terminal lucidity in patients with chronic schizophrenia and dementia: A survey of the literature. *J. Nerv. Ment. Dis.* 197 942–944. 10.1097/NMD.0b013e3181c22583 20010032

[B88] Nahm M. Greyson B. Kelly E. W. Haraldsson E. (2012). Terminal lucidity: A review and a case collection. *Arch. Gerontol. Geriatr.* 55 138–142. 10.1016/j.archger.2011.06.031 21764150

[B89] Neppe V. M. Close E. R. (2015). Refuting atomic materialism. *IQNexus* 11 1074–1083.

[B90] Neppe V. M. Close E. R. (2020). The Neppe-Close triadic dimensional vortical paradigm: An invited summary. *Int. J. Phys. Res. Appl.* 3 001–004. 10.29328/journal.ijpra.1001018

[B91] Oizumi M. Albantakis L. Tononi G. (2014). From the phenomenology to the mechanisms of consciousness: Integrated information theory 3.0. *PLoS Comput. Biol.* 10:e1003588. 10.1371/journal.pcbi.1003588 24811198 PMC4014402

[B92] Palmer J. (1979). A community mail survey of psychic experiences. *J. Am. Soc. Psych. Res.* 73 221–251.

[B93] Palmer J. Millar B. (2015). “Experimenter effects in parapsychological research,” in *Parapsychology: A handbook for the 21st century*, eds Cardeña E. Palmer J. Marcusson-Clavertz D. (Jefferson, NC: McFarland & Company, Inc., Publishers), 293–300.

[B94] Planck M. (1950). in *Scientific autobiography and other papers*, ed. Gaynor F. (London: Williams & Northgate).

[B95] Radin D. I. (1997). Unconscious perception of future emotions: An experiment in presentiment. *J. Sci. Explor.* 11 163–180.

[B96] Radin D. I. Stone J. Levine E. Eskandarnejad S. Schlitz M. Kozak L. (2008). Compassionate intention as a therapeutic intervention by partners of cancer patients: Effects of distant intention on the patients’ autonomic nervous system. *Explore J. Sci. Heal.* 4 235–243. 10.1016/j.explore.2008.04.002 18602616

[B97] Radin D. I. Vieten C. Michel L. Delorme A. (2011). Electrocortical activity prior to unpredictable stimuli in meditators and non-meditators. *Explore* 7 286–299. 10.1016/j.explore.2011.06.004 21907152

[B98] Radin D. Borges A. (2009). Intuition through time: What does the seer see? *Explore* 5 200–211. 10.1016/j.explore.2009.04.002 19608110

[B99] Radin D. Lobach E. (2007). Toward understanding the placebo effect: Investigating a possible retrocausal factor. *J. Altern. Complement. Med.* 13 733–739. 10.1089/acm.2006.6243 17931066

[B100] Radin D. Pierce A. (2015). “Psi and Psychophysiology,” in *Parapsychology: A handbook for the 21st century*, ed. Cardena E. (Jefferson, NC: McFarland & Company, Inc., Publishers).

[B101] Rao R. P. N. Ballard D. H. (1999). Predictive coding in the visual cortex: A functional interpretation of some extra-classical receptive-field effects. *Nat. Neurosci.* 2 79–87. 10.1038/4580 10195184

[B102] Reardon S. (2019). *‘Outlandish’ competition seeks the brain’s source of consciousness.* Available online at: https://www.science.org/content/article/outlandish-competition-seeks-brain-s-source-consciousness (accessed May 16, 2022).

[B103] Richards T. L. Kozak L. Johnson L. C. Standish L. J. (2005). Replicable functional magnetic resonance imaging. Evidence of correlated brain signals between physically and sensory isolated subjects. *J. Altern. Complement. Med.* 11 955–963. 10.1089/acm.2005.11.955 16398586

[B104] Ross C. A. Joshi S. (1992). Paranormal experiences in the general population. *J. Nerv. Ment. Dis.* 180 357–361. 1593270 10.1097/00005053-199206000-00004

[B105] Saxe J. G. (2016). *The blind men and the elephant.* Hong Kong: Enrich Spot Limited.

[B106] Schlitz M. Wiseman R. Watt C. Radin D. (2006). Of two minds: Sceptic-proponent collaboration within parapsychology. *Br. J. Psychol.* 97(Pt 3), 313–322. 10.1348/000712605X80704 16848945

[B107] Schmidt S. (2012). Can we help just by good intentions? A meta-analysis of experiments on distant intention effects. *J. Altern. Complement. Med.* 18 529–533. 10.1089/acm.2011.0321 22784339

[B108] Schmidt S. (2015). “Experimental research on distant intention phenomena,” in *Parapsychology: A handbook for the 21st century*, eds Cardeña E. Palmer J. Marcusson-Clavertz D. (Jefferson, NC: McFarland & Company, Inc., Publishers), 244–257. 10.1016/j.explore.2015.10.001

[B109] Schmidt S. Schneider R. Utts J. Walach H. (2004). Distant intentionality and the feeling of being stared at: Two meta-analyses. *Br. J. Psychol.* 95 (Pt 2), 235–247. 10.1348/000712604773952449 15142304

[B110] Schooler J. (2014). “Bridging the objective/subjective divide: Towards a meta-perspective of science and experience,” in *Open MIND*, (Frankfurt: Frankfurt am Main: MIND Group).

[B111] Schwartz S. A. (2005). *The secret vaults of time: Psychic archaeology and the quest for man’s beginnings*, Vol. 12. Newburyport, MA: Hampton Roads Publishing.

[B112] Schwartz S. A. (2019). The location and reconstruction of a byzantine structure in marea, egypt, including a comparison of electronic remote sensing and remote viewing. *J. Sci. Explor.* 33 451–480.

[B113] Selimbeyoglu A. Parvizi J. (2010). Electrical stimulation of the human brain: Perceptual and behavioral phenomena reported in the old and new literature. *Front. Hum. Neurosci.* 4:46. 10.3389/fnhum.2010.00046 20577584 PMC2889679

[B114] Seth A. K. (2013). Interoceptive inference, emotion, and the embodied self. *Trends Cogn. Sci.* 17 565–573. 10.1016/j.tics.2013.09.007 24126130

[B115] Seth A. K. Bayne T. (2022). Theories of consciousness. *Nat. Rev. Neurosci.* 23 439–452. 10.1038/s41583-022-00587-4 35505255

[B116] Shapiro K. L. Raymond J. E. Arnell K. M. (1997). The attentional blink. *Trends Cogn. Sci.* 1 291–296. 10.1016/S1364-6613(97)01094-2 21223931

[B117] Simons D. J. Chabris C. F. (1999). Gorillas in our midst: Sustained inattentional blindness for dynamic events. *Perception* 28 1059–1074. 10.1068/p281059 10694957

[B118] Smith C. C. Laham D. Moddel J. (2014). Stock market prediction using associative remote viewing by inexperienced remote viewers. *J. Sci. Explor.* 28 7–16.

[B119] Standish L. J. Johnson L. C. Kozak L. Richards T. (2003). Evidence of correlated functional magnetic resonance imaging signals between distant human brains. *Altern. Ther.* 9 122–125. 14640097

[B120] Standish L. J. Kozak L. Johnson L. C. Richards T. (2004). Electroencephalographic evidence of correlated event-related signals between the brains of spatially and sensory isolated human subjects. *J. Altern. Complement. Med.* 10 307–314. 10.1089/107555304323062293 15165411

[B121] Steriade M. Timofeev I. Grenier F. (2001). Natural waking and sleep states: A view from inside neocortical neurons. *J. Neurophysiol.* 85 1969–1985. 10.1152/jn.2001.85.5.1969 11353014

[B122] Stevenson I. Pasricha S. (1979). A case of secondary personality with xenoglossy. *Am. J. Psychiatry* 136 1591–1592. 10.1176/ajp.136.12.1591 507214

[B123] Stevenson I. Pasricha S. (1980). A preliminary report on an unusual case of the reincarnation type with xenoglossy. *J. Am. Soc. Psych. Res.* 74 331–348.

[B124] Storm L. Tressoldi P. (2020). Meta-analysis of free-response studies 2009-2018: Assessing the noise-reduction model ten years on. *J. Soc. Psych. Res.* 84 193–219. 10.1037/a0019457 20565164

[B125] Storm L. Tressoldi P. E. Di Risio L. (2010). Meta-analysis of free-response studies, 1992-2008: Assessing the noise reduction model in parapsychology. *Psychol. Bull.* 136 471–485. 10.1037/a0019457 20565164

[B126] Storm L. Tressoldi P. E. Di Risio L. (2012). Meta-analysis of ESP studies, 1987-2010: Assessing the success of the forced-choice design in parapsychology. *J. Parapsychol.* 76 243–273.

[B127] Templeton World Charity Foundation. (2022). *Accelerating research on consciousness. Templeton world charity foundation.* Available online at: https://www.templetonworldcharity.org/our-priorities/accelerating-research-consciousness (accessed May 19, 2022).

[B128] Tononi G. (2015). Integrated information theory. *Scholarpedia* 10:4164. 10.4249/scholarpedia.4164

[B129] Tononi G. Boly M. Massimini M. Koch C. (2016). Integrated information theory: From consciousness to its physical substrate. *Nat. Rev. Neurosci.* 17 450–461. 10.1038/nrn.2016.44 27225071

[B130] Treffert D. A. (2009). The savant syndrome: An extraordinary condition. A synopsis: Past, present, future. *Philos. Trans R. Soc. Lond. B. Biol. Sci.* 364 1351–1357. 10.1098/rstb.2008.0326 19528017 PMC2677584

[B131] Tressoldi P. Martinelli M. Zaccaria E. Massaccesi S. (2009). Implicit intuition: How heart rate can contribute to predict future events. *J. Soc. Psych. Res.* 73 1–16.

[B132] Utts J. (2016). Appreciating Statistics. *J. Am. Stat. Assoc.* 111 1373–1380. 10.1080/01621459.2016.1250592

[B133] Vandersande J. W. (2008). *Life after death: Some of the best evidence.* Denver: Outskirts Press, Inc.

[B134] VanRullen R. Kanai R. (2021). Deep learning and the Global Workspace Theory. *Trends Neurosci.* 44, 692–704. 10.1016/j.tins.2021.04.005 34001376

[B135] Wahbeh H. Radin D. Mossbridge J. Vieten C. Delorme A. (2018). Exceptional experiences reported by scientists and engineers. *Explore* 14 329–341. 10.1016/j.explore.2018.05.002 30415782

[B136] Watt C. Wiseman R. Schlitz M. (2002). Tacit information in remote staring research: The Wiseman-Schlitz interviews. *Paranormal Rev.* 24 18–25.

